# Non-Redundant Role for IL-12 and IL-27 in Modulating Th2 Polarization of Carcinoembryonic Antigen Specific CD4 T Cells from Pancreatic Cancer Patients

**DOI:** 10.1371/journal.pone.0007234

**Published:** 2009-10-02

**Authors:** Elena Tassi, Marco Braga, Renato Longhi, Francesca Gavazzi, Giorgio Parmiani, Valerio Di Carlo, Maria Pia Protti

**Affiliations:** 1 Tumor Immunology Unit, San Raffaele Scientific Institute, Milan, Italy; 2 Pancreas Unit, San Raffaele Scientific Institute, Milan, Italy; 3 Immuno-Biotherapy of Melanoma and Solid Tumors Unit, San Raffaele Scientific Institute, Milan, Italy; 4 Università Vita-Salute San Raffaele, Milan, Italy; 5 Division of Immunology, Transplantation and Infectious Diseases, San Raffaele Scientific Institute, Milan, Italy; 6 Division of Molecular Oncology, San Raffaele Scientific Institute, Milan, Italy; 7 Department of Surgery, San Raffaele Scientific Institute, Milan, Italy; 8 CNR-Istituto di Chimica del Riconoscimento Molecolare, Milan, Italy; New York University School of Medicine, United States of America

## Abstract

**Background:**

Pancreatic cancer is a very aggressive disease with dismal prognosis; peculiar is the tumor microenvironment characterized by an extensive fibrotic stroma, which favors rapid tumor progression. We previously reported that pancreatic cancer patients have a selective Th2 skew in the anti-carcinoembryonic antigen (CEA) CD4^+^ T cell immunity, which correlates with the presence of a predominant GATA-3^+^ tumor lymphoid infiltrate. This has negative effects in both effective anti-tumor immunity and further favoring fibrinogenesis. Aim of this study was to evaluate whether the Th2 polarization of CEA-specific CD4^+^ T cells from pancreatic cancer patients is stable or can be reverted by immunomodulating cytokines.

**Methodology/Principal Findings:**

We first evaluated the influence of IL-12 and IL-27, as single agents and in association, on the polarization of CEA-specific Th2 CD4^+^ T cell clones from a pancreatic cancer patient. We found that only the combination of IL-12 and IL-27 modified the polarization of Th2 effectors by both reduction of IL-5, GM-CSF and IL-13 and induction of IFN-γ production, which lasted after cytokine removal. Second, we evaluated the effect of the combined treatment on polyclonal CEA-specific CD4^+^ T cells in short-time re-stimulation assays. In agreement with the data obtained with the clones, we found that the combined treatment functionally modulated the Th2 polarization of CEA-specific CD4^+^ T cells and enhanced pre-existing Th1 type immunity.

**Conclusions/Significance:**

Collectively, our results demonstrate that tumor antigen specific Th2 CD4^+^ T cells in pancreatic cancer are endowed with functional plasticity. Hence, loco-regional cytokines delivery or targeted therapy based on antibodies or molecules directed to the tumor stroma might improve anti-tumor immunity and ameliorate fibrosis, without systemic toxicity.

## Introduction

Pancreatic cancer (PC) is a very aggressive disease with dismal prognosis [1]. Peculiar is the tumor microenvironment, which consists of fibroblasts, pancreatic stellate cells, endothelial cells, immune and endocrine cells and perineural spreading and it is believed to play an active role in disease progression and aggressiveness [2].

Recently [3], we investigated the quality of the anti-tumor CD4^+^ T cell responses in PC patients undergoing surgical resection, by comparing the anti-carcinoembryonic (CEA) and anti-viral CD4^+^ T cell immunity. We found that anti-CEA CD4^+^ T cell immunity was present in a significantly lower number of PC patients compared to normal donors. Most importantly, while CD4^+^ T cells from normal donors produced mainly granulocytes macrophages colony stimulating factor (GM-CSF) and the pro-inflammatory (*i.e.*, Th1) cytokine interferon-γ (IFN-γ), CD4^+^ T cells from the patients produced mainly interleukin(IL)-5 and IL-13, demonstrating a skew towards an anti-inflammatory (*i.e.*, Th2) type. On the contrary, the extent of anti-viral CD4^+^ T cell immunity was comparable between the two groups and showed a Th1 type. In agreement with the Th2 skew observed in circulating CEA specific CD4^+^ T cells, immunohistochemical analysis of tumor infiltrating lymphocytes showed a significantly higher number of GATA-3^+^ (Th2) compared to T-bet^+^ (Th1) lymphoid cells, supporting a Th2 skew also at the tumor site.

It has been shown that fibrosis, which is a hallmark in PC, is strongly linked to the development of Th2 responses through activation by Th2 cytokines of collagen synthesis by fibroblasts and concomitant reduced collagen degradation in the absence of Th1 cytokines [4]. Therefore, the presence of GATA-3^+^ lymphoid cells, which we observed [3] in the pancreatic tumor microenvironment, might further contribute to fibrosis and local treatments aimed at reducing the presence of Th2 cytokines might be beneficial.

Among the various factors that promote CD4^+^ T cells polarization, cytokines have a determinant role [5]. IL-12, a product of phagocytes and dendritic cells in response to microbial stimulation, has a major role in promoting Th1 polarization and in enhancing CD4^+^ T cells proliferation [6]. Moreover, IL-12 has been shown to induce reversal of Th2 polarization in human allergen-specific Th2 cells [7], [8] and, in synergy with IL-18, to stimulate IFN-γ production of peripheral blood mononuclear cells (PBMC) from lepromatous leprosy patients [9]. IL-27, a recently identified member of the IL-12 family [10], also stimulates proliferation and synergizes with IL-12 in triggering IFN-γ production of naïve CD4^+^ T cells [10], [11]. More recently, it has been shown [12] that IL-27 inhibits Th2 polarization of naïve murine CD4^+^ T cells and suppresses Th2 cytokines production from *in vitro* polarized Th2 cells.

In the present study we investigated the possibility to revert the Th2 polarization of *bona fide in vivo* primed CEA-specific CD4^+^ T cells from PC patients by using IL-12 and IL-27 as immunomodulating agents. We found that the presence of IL-27 alone was sufficient to inhibit IL-5 and IL-13 secretion, while IL-12 strongly stimulated IFN-γ production with minor effects on Th2 cytokines secretion; the combination of the two cytokines induced a functional modulation of the Th2 polarization.

## Materials and Methods

### Subjects and cell lines

PBMC were obtained from two PC patients (pt#15 and pt#43) and one healthy donor (ND#11) of a cohort of subjects in which we previously detected anti-CEA CD4^+^ T cells [3]. Patients were drawn before surgery and staging of the disease was T3N1M0 in both cases. The Institutional Ethics Committee (Comitato Etico Fondazione Centro San Raffaele del Monte Tabor, Istituto Scientifico Ospedale San Raffaele) had approved the study protocol and written informed consent was obtained from all donors before blood sampling. EBV-transformed lymphoblastoid cell lines (LCL) used and their human leukocyte antigen (HLA)-DR type were: SKP-LCL (β1*0405, *1401; β3*02), established in our laboratory from a healthy donor; BM21 (β1*1101; β3*0202) and Pitout (β1*0701; β4*0101), kindly provided by K. Fleischhauer (San Raffaele Scientific Institute, Milan). LCL were cultured in RPMI 1640 (BioWhittaker) containing 2 mM L-glutamine, 100 units/ml penicillin, 50 mg/ml streptomycin (BioWhittaker), and 10% FCS (BioWhittaker).

### Synthesis of CEA peptides

CEA_99–111_, CEA_117–129_, CEA_177–189/355–367_, CEA_425–437_, CEA_568–582_, and CEA_666–678_ sequences were synthesized by the stepwise solid-phase method as previously described [13]; the peptides were lyophilized, reconstituted in DMSO (Sigma-Aldrich) at 10 mg/ml and diluted in RPMI 1640 as needed.

### *In vitro* propagation of CD4^+^ T cell clones

Polyclonal CEA_177–189/355–367_ specific CD4^+^ T cells from pt#15, obtained after 13 days of short-term culture with the specific peptide and autologous CD4^+^-depleted PBMC as antigen presenting cells (APC), were cloned by limiting dilution as described in [14]. The clones were cultured in X-VIVO 15 (BioWhittaker) supplemented with 5% heat-inactivated pooled human serum (BioWhittaker), penicillin (100 U/ml; BioWhittaker), streptomycin (50 mg/ml; BioWhittaker), and IL-2 (250 IU/ml; Proleukine, Novartis). Clones were re-stimulated every 15–21 days with phytohemagglutinin (PHA) (0,5 µg/ml; Sigma-Aldrich) and irradiated allogenic PBMC.

### CD4^+^ T cell clones stimulation assay

CD4^+^ T cells (10^4^/well) were cultured in triplicate in 96-U bottom plates in the presence of irradiated LCL (5×10^4^/well), or irradiated autologous or HLA-DRβ3*02 matched PBMC (10^5^/well) as APC pulsed with the CEA_177–189/355–367_ peptide (10 µg/ml), or the purified CEA protein (30 µg/ml, BiosPacific), or normal human IgG (30 µg/ml, Venimmun N, Aventis Behring), as negative control. In inhibition experiments the following monoclonal antibodies (mAbs) (Beckton Dickinson) were used: anti-HLA-DR (100 ng/ml), anti-HLA-DP (3 µg/ml) and anti-HLA-DQ (125 ng/ml). In peptide titration experiments, the following concentrations of peptide were added: 20-10-5-1-0,5-0,1-0,05-0,01 and 0,005 µg/ml. In the experiments with immunomodulatory cytokines, recombinant human IL-12 and/or IL-27 (R&D Systems Inc.) were added at the following concentrations: IL-12, as single agent (5–20 ng/ml); IL-27, as single agent (0,01-1-10-100 ng/ml); combined IL-12 and IL-27 treatment (5 ng/ml+100 ng/ml, respectively). After 48 h, supernatant from each well was removed and pooled for cytokines' detection by ELISA, according to the manufacturers' instructions: GM-CSF (sensitivity threshold: 31,25 pg/ml) and transforming growth factor (TGF)-β1 (sensitivity threshold: 62,5 pg/ml) (Biosource International Inc.); IFN-γ, IL-5 and IL-13 (sensitivity threshold: 15,6 pg/ml) (Mabtech) and IL-17 (eBioscience) (sensitivity threshold: 7,8 pg/ml). IFN-γ, tumor necrosis factor (TNF)-α, IL-10, IL-4, IL-5 and IL-2 release was also determined by using the Cytometric Beads Array (CBA) Human Th1-Th2 Cytokines Kit (sensitivity threshold: 20 pg/ml), (Becton Dickinson), following the manufacturer's instructions.

### Short-term culture and cytokine release assay

Purified CD4^+^ T cells were stimulated once *in vitro* in the presence of the CEA peptides as previously described [3]. Briefly, CD4^+^ T cells (3×10^4^/well), purified from total PBMC by magnetic beads (Miltenyi Biotec), were plated in 96-well plates in five replicates for each condition and cultured in X-VIVO 15 supplemented with penicillin (100 U/ml), streptomycin (50 mg/ml) and 3% heat-inactivated pooled human serum (tissue culture medium, TCM), in the presence of irradiated CD4^+^-depleted PBMC as APC, at a CD4^+^: APC ratio of 1∶3. Stimuli were: PHA (10 µg/ml; Sigma-Aldrich), as positive control; CD4^+^ T cells in the presence of the APC only, as baseline (blank); and each single peptide (10 µg/ml). Recombinant human IL-12 (5 ng/ml) and IL-27 (100 ng/ml) were added where indicated. At day 7, half medium from each well was removed and replenished with fresh TCM containing IL-2 (25 IU/ml), and IL-12 and IL-27 where indicated, without any further antigen stimulation. At day 14, supernatant was collected for IFN-γ, IL-5, IL-13 and GM-CSF release by ELISA, as described above.

### Flow cytometry

Cytofluorimetric analyses were performed on a FACSCalibur and analyzed using the FlowJo software (Tree Star, Inc.). The following antibodies were used: anti-CD3-APC, anti-CD4-PercP, anti-CCR4-PE, anti-CCR5-FITC (Pharmingen) and anti-CRTH2-PE (Miltenyi Biotec). TCRVβ expression was determined with the IO Test Beta Mark kit (Immunotech), following the manufacturer's instruction.

## Results

### Characterization of CEA_177–189/355–367_ specific Th2-cytokines producing CD4^+^ T cell clones

**Figure 1:**
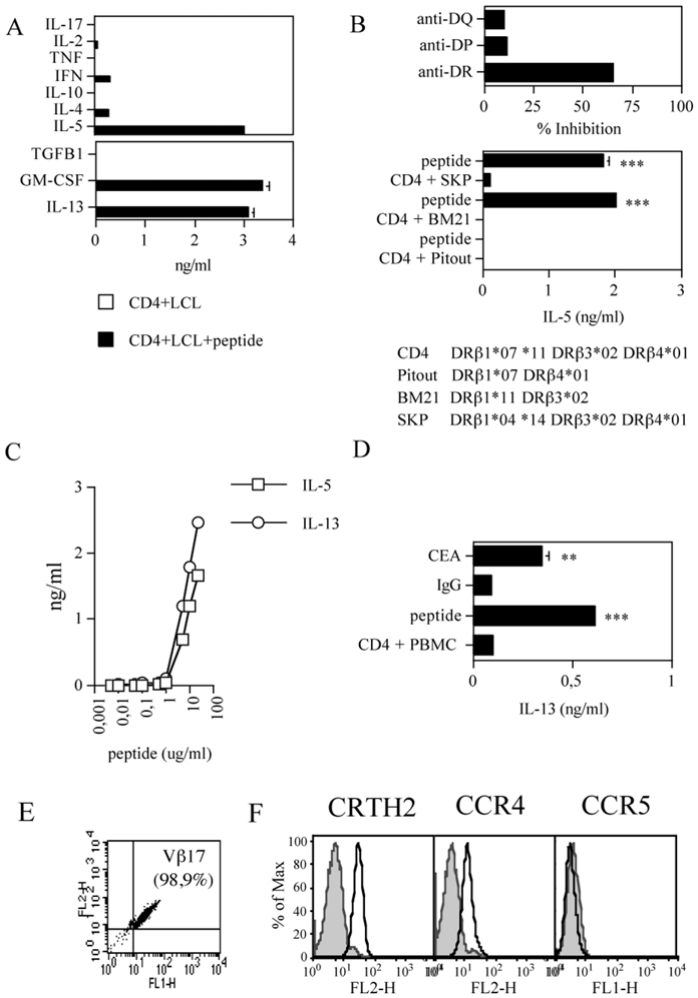
Characterization of CEA_177–189/355–367_ specific CD4^+^ T cell clones from pt#15. *Profile of cytokine secreted (A)*. CD4^+^ T cells were cultured with irradiated APC in the presence or the absence of the relevant peptide in a 2-day stimulation assay and the concentration of the indicated cytokines in the supernatants was determined either by CBA (upper panel) or ELISA (lower panel). The data are representative of at least six experiments; for ELISA assays, the data are means of duplicate determination±SD. *HLA restriction (B)*. CD4^+^ T cells were cultured with irradiated autologous PBMC or HLA-DR matched LCL, as indicated, in the presence or the absence of the relevant peptide (10 µg/ml) and in the absence or the presence of anti-HLA-DR, anti-HLA-DP and anti-HLA-DQ mAbs: after 2 days IL-5 was tested. Upper panel: % inhibition was calculated based on IL-5 secretion by CD4^+^ T cells in the presence of the relevant peptide (2 ng/ml over 0 ng/ml of background level). The data are representative of two (upper panel) and four (lower panel) experiments and are means of duplicate determination±SD. Responses significantly higher than the blanks (*i.e.*, the basal levels of cytokines secretion from CD4^+^ T cells in the presence of LCL only) are indicated as: ***, p<0.001 (determined by unpaired, one-tailed Student's t test). *Dose-response curves (C)*. CD4^+^ T cells were cultured with titrated doses of the relevant peptide in the presence of irradiated APC in a 2-day stimulation assay and tested for IL-5 and IL-13 release. The data are means of duplicate determination±SD. *Recognition of the native protein (D)*. CD4^+^ T cells were cultured in the presence of irradiated PBMC, as APC, pulsed with either the relevant peptide (10 µg/ml) as positive control, or the purified CEA protein (30 µg/ml) or human IgG (30 µg/ml) as negative control in a 2-day stimulation assay and tested for IL-13 release. The data are means of duplicate determination±SD. Responses significantly higher than the blanks (*i.e.*, the basal levels of cytokines secretion from CD4^+^ T cells in the presence of PBMC only) are indicated as: **, 0.001<p<0.05; ***, p<0.001 (determined by unpaired, one-tailed Student's t test). *TCRVβ expression (E)*. The test was performed with the IO Test Beta Mark kit. Quadrants were set based on isotype control staining. *Surface expression of Th2 (CRTH2 and CCR4) and Th1 (CCR5) markers (F)*. Filled histograms represent isotype controls; open histograms samples stained with the indicated markers.

We previously reported that PC patients have a Th2 skew in anti-CEA CD4^+^ T cell immunity while maintaining an intact repertoire of anti-viral Th1 cells [3]. To better characterize the feature of this anti-CEA response we cloned by limiting dilution CEA_177–189/355–367_ specific polyclonal CD4^+^ T cells from pt #15, obtained after an *in vitro* short-term re-stimulation of CD4^+^ T cells with autologous APC pulsed with the relevant peptide; an assay that we previously showed not to favor *in vitro* priming [15]. Polyclonal cells produced high levels of IL-5 and very little of GM-CSF but no IFN-γ [3], suggesting the presence of *in vivo* primed CD4^+^ T cells with Th2 features. Two clones were obtained, which expressed the same T cell receptor (TCR) Vβ chain, *i.e.* the Vβ17 (Fig. 1E), and therefore we used them indifferently in the following experiments being *bona fide* the same clone.

The characterization of the CEA_177–189/355–367_ specific CD4^+^ T clones is depicted in Figure 1. CD4^+^ T cell clones produced mainly IL-5, IL-13 and GM-CSF, while they released low amount of IL-4, and IFN-γ and no IL-2, TNF-α, TGF-β1, IL-10 or IL-17 (Fig. 1A). Although the cells produce little amount of IL-4, this pattern of cytokine secretion is consistent with a Th2 polarization.

To identify the restriction element we first tested the recognition by CD4^+^ T cells of peptide loaded autologous APC in the absence or in the presence of anti-HLA-DR, anti-HLA-DP and anti-HLA-DQ mAbs. As shown in Fig. 1B (upper panel), addition of the anti-DR mAb inhibited (65,5%) IL-5 production by CD4^+^ T cells, demonstrating that an HLA-DR molecule presented the peptide. To identify the HLA-DR presenting allele, the cells were then challenged with the relevant peptide in the presence of LCL expressing different combinations of the HLA-DRβ1, β3 and β4 alleles expressed by the patient (indicated in Fig. 1B) and tested for IL-5 release. As shown in Fig. 1B (lower panel), CD4^+^ T cells recognized the peptide in association with HLA-DRβ3*02, which is the allele shared between the patient and the two presenting LCL (BM21 and SKP).

We also performed peptide titration curve in which CD4^+^ T cells were challenged with increasing concentration of the relevant peptide (Fig. 1C). The experiments indicated the expression by the clone of a very low affinity TCR.

We previously showed that CEA sequence repeated at positions 177–189 and 355–367 contains an *in vitro* naturally processed epitope presented in association with several HLA-DR alleles [16]. To confirm these data, CD4^+^ T cell clones were challenged with the CEA protein or normal human IgG, as a negative control, and assayed for IL-13 release. As shown in Fig. 1D, CD4^+^ T cells specifically produced IL-13 in the presence of the peptide and, most importantly, in the presence of the CEA but not of the control protein (*i.e.*, human IgG), demonstrating that although CEA_177–189/355–367_ specific CD4^+^ T cell clones carry a low affinity TCR they recognize the native epitope.

Finally, to verify whether the Th2 cytokine profile correlated with the phenotypic markers of Th1 and Th2 cells, the clones were tested for the expression of CCR5, which is preferentially expressed by Th1 cells [17] and of CRTH2 and CCR4, whose expression characterizes Th2 cells [17], [18]. As expected from the cytokine profile, CD4^+^ T cells expressed on their surface both CRTH2 and CCR4 while CCR5 expression was absent (Fig. 1F).

### Combined IL-27 and IL-12 treatment inhibits secretion of Th2 cytokines and stimulates IFN-γ production by CEA specific CD4^+^ T cells

We next tested the possibility to modulate the polarization of the CEA_177–189/355–367_-specific Th2 cells by the immunomodulatory cytokines IL-27 and IL-12.

**Figure 2:**
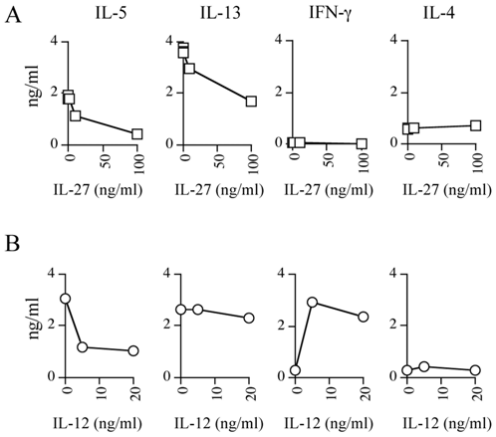
Effect of titrated doses of IL-12 or IL-27 as single agent on the repertoire of cytokine secreted by CEA_177–189/355–367_ specific CD4^+^ T cells. *(A).* CD4^+^ T cells were cultured with the relevant peptide and irradiated LCL as APC in the presence of increasing concentrations of IL-27 (0-0,1-1-10-100 ng/ml); after 2 days the cytokine release was evaluated by CBA (IL-5 IL-4, and IFN-γ) or ELISA (IL-13). The data for IL-13 are means of duplicate determination±SD. *(B).* CD4^+^ T cells were cultured and tested, as described above, in the presence of increasing concentrations of IL-12 (0-5-20 ng/ml). The basal level of cytokines secretion of CD4^+^ T cells in the presence of LCL only was subtracted from the sample values and was comprised between 0 and 0,018 ng/ml. The data are representative of at least three experiments.

To this aim we first evaluated the effect of increasing concentrations of IL-27 or IL-12, as single agents, on the production by CD4^+^ T cells of Th1 and Th2 cytokines in the presence of the relevant peptide. The addition of IL-27 decreased in a dose dependent-manner IL-5 and IL-13 production, while no effect was observed for IL-4 and IFN-γ (Fig. 2A). The addition of IL-12 reached its plateau effect already at the dose of 5 ng/ml (Fig. 2B): IL-5 production decreased also in the presence of IL-12 while, at difference with IL-27, the effect on IL-13 was marginal as on IL-4. Importantly, and at difference with IL-27, IFN-γ production was strongly increased (Fig. 2B). These data demonstrate that both cytokines affect the effector function of the CEA-specific CD4^+^ T cell clones but neither IL-27 nor IL-12, as single agents, were optimal in modulating their Th polarization.

**Figure 3:**
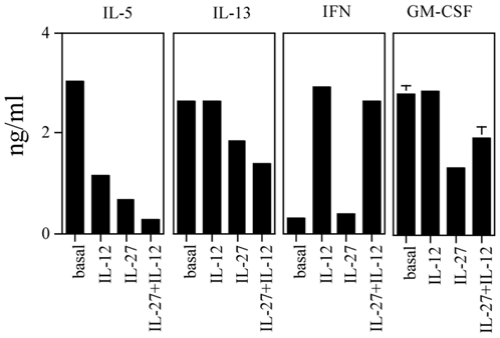
Modulation of Th2 polarization of CEA_177–189/355–367_ specific CD4^+^ T cells by combined IL-12 and IL-27 treatment. CD4^+^ T cells were cultured with the relevant peptide in the absence (basal) or in the presence of IL-12 (5 ng/ml), as single agent; or IL-27 (100 ng/ml), as single agent; or combined IL-12 and IL-27 in a 2-day stimulation assay and then tested for cytokine release by CBA (IL-5 and IFN-γ) or ELISA (IL-13 and GM-CSF). The data for IL-13 and GM-CSF are means of duplicate determination±SD. The basal level of cytokines secretion of CD4^+^ T cells in the presence of LCL only was subtracted from the sample values and was comprised between 0 and 0,006 ng/ml. The data are representative of five experiments.

Second, we evaluated the effect of the combined treatment with the two cytokines (Fig. 3). When used at 5 ng/ml, IL-12, as a single agent, induced 61% inhibition of IL-5 and had no effect on IL-13 and GM-CSF, while IFN-γ production had almost a 10-fold increase. IL-27 treatment alone at the highest concentration showed 78%, 30% and 53% inhibition of IL-5, IL-13 and GM-CSF, respectively; while only 1,4-fold increase in IFN-γ production. When used in combination at the best concentrations, a synergistic effect was observed in the inhibition of IL-5 (91%) and IL-13 (48%), while, as expected from the results of the single agents, GM-CSF secretion was not further inhibited and IFN-γ production was not further increased.

### Modulation of Th2 polarization by IL-12 and IL-27 lasts after cytokines removal

**Figure 4:**
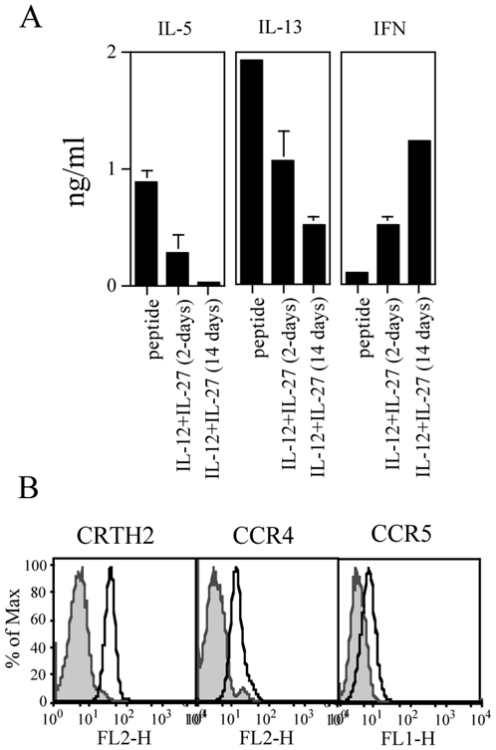
Effects of combined IL-12 and IL-27 given as a single shot or continuously in culture. *(A)*. CD4^+^ T cells were cultured with the relevant peptide and either left untreated (peptide) or treated for 2 days with combined IL-12 (5 ng/ml) and IL-27 (100 ng/ml) and then washed and left untreated (IL-12+IL-27 (2-days)) or treated continuously with IL-12 and IL-27 at the same doses for 2 weeks (IL-12+IL-27 (14 days)). At day 14, cells were tested in the presence or the absence of the relevant peptide and the specific IL-5, IL-13 and IFN-γ release in the supernatant tested by ELISA. The data are means of duplicate determination±SD. The basal level of cytokines secretion of CD4^+^ T cells in the presence of LCL only was subtracted from the sample values and was as follows: IL-5 (0,093±0,006 ng/ml), IL-13 (0,468±0,028 ng/ml) and IFN-γ (0 ng/ml). The data are representative of four experiments. (*B*). Surface expression of CRTH2, CCR4 CCR5 by CD4^+^ T cells after treatment with combined IL-12+IL-27. Analysis was performed on cells treated for 2 days and then left untreated for a further week. Filled histograms represent isotype controls; open histograms samples stained with the indicated markers.

To verify whether the modulation of Th2 polarization obtained by the combined treatment with IL-12 and IL-27 was stable, we designed an experiment in which CEA-specific CD4^+^ T cells were first stimulated with the relevant peptide in the absence or in the presence of IL-12 and IL-27 (5 and 100 ng/ml, respectively). Second, cells stimulated in the absence of the cytokines were kept in culture for 14 days in TCM plus IL-2 and used as controls. Cells treated with the cytokines were divided in two aliquots and cultured under different conditions for the following 14 days: in one condition the cytokines were removed after 2 days and the cells cultured as the controls in TCM plus IL-2, in the other condition cytokines were kept in culture all the time and replaced every two to three days. At day 14, cells from each of the three conditions were tested in a 2-day stimulation assay with the relevant peptide and cytokine release tested. The results obtained are shown in Figure 4. Control cells confirmed production of IL-5, IL-13 and no IFN-γ, while cells kept in culture continuously with the cytokines confirmed 98% and 74% inhibition of IL-5 and IL-13 production, respectively, and a 11-fold increase for IFN-γ. Importantly, cells in which cytokines were removed after 2 days of culture still showed 70% and 47% inhibition in IL-5 and IL-13 production, respectively, and a 5-fold increase in IFN-γ release.

To verify whether the combined treatment also influenced the Th2 surface phenotype, CD4^+^ T cells, which had been re-stimulated in the presence of the cytokines for 2 days and then cultured for further seven days in the absence of the cytokines, were tested for the expression of CRTH2, CCR4 and CCR5. As shown in Figure 4B, the cells, while maintaining CRTH2 and CCR4 as in the absence of treatment (Fig. 1F), acquired the expression of CCR5.

Collectively, these results show that modulation of Th2 polarization of CEA-specific CD4^+^ T cell clones obtained with the combined treatment is lasting, although with reduced efficiency, even after cytokines removal and is associated with functional and phenotypic features of Th0 or both Th1 and Th2 cells.

### IL-12 and IL-27 combination enhances Th1 and modulates Th2 polarization of spontaneous anti-CEA CD4^+^ T cell responses

**Figure 5:**
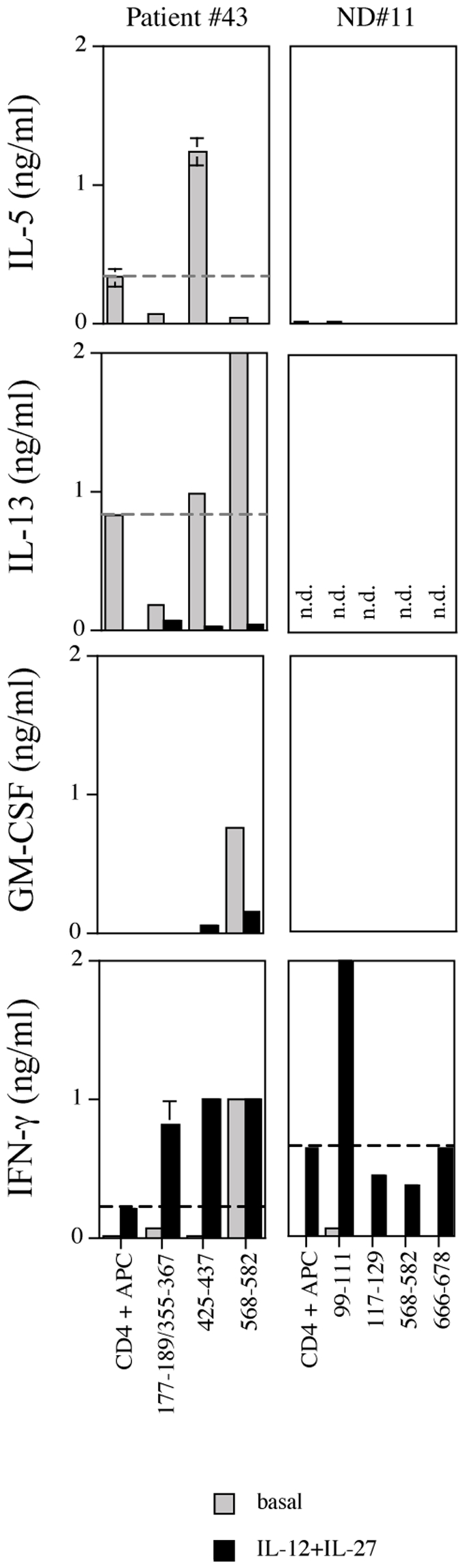
Combined treatment with IL-12 and IL-27 modulates polarization of Th2 and enhances IFN-γ production by pre-existing Th1 anti-CEA CD4^+^ T cells. CD4^+^ T cells from pt#43 and ND#11 were cultured in five replicates, as described in Materials and Methods, with the responsive CEA peptides, in the absence (grey bars) or in the presence (black bars) of the combined treatment with IL-12 (5 ng/ml) plus IL-27 (100 ng/ml). After 14 days, IL-5, IL-13, GM-CSF and IFN-γ release was tested by ELISA. Data reported are means of duplicate determination±SD. Dashed lines identify the basal level of cytokine secretion in the presence of APC only. n.d. = not determined.

To verify the effect of combined IL-12 and IL-27 treatment on the polarization of CEA-specific CD4^+^ T cells in a more physiologic environment and at polyclonal level, CD4^+^ T cells from patient (pt#43) and normal donor (ND#11) were tested in a 14-days *in vitro* re-stimulation assay for the recognition of the relevant peptides in the absence or in the presence of the two immunomodulatory cytokines (Figure 5). As previously reported in [3], in the absence of the immunomodulatory cytokines CD4^+^ T cells from pt#43 produced IL-5 in the presence of CEA_425–437_; IL-13 in the presence of CEA_568–582_; GM-CSF in the presence of CEA_568–582_ and IFN-γ in the presence of CEA_568–582_ and, although at a much lower but significant level, of CEA_177–189/355–367_ (Fig. 5, *left panel* *s, grey bars*). The combination of IL-12 and IL-27 almost abolished IL-5 (99% inhibition) and IL-13 (76% inhibition) while induced *de novo* IFN-γ secretion in the presence of CEA_425–437_, enhanced (9-fold increase) IFN-γ production in the presence of CEA_177–189/355–367_ and confirmed IFN-γ while strongly reduced IL-13 (98% inhibition) and GM-CSF (80% inhibition) production in the presence of CEA_568–582_ (Fig. 5, *left panel* *s, black bars*). As previously reported [3], in the absence of the immunomodulatory cytokines CD4^+^ T cells from ND#11 showed significant proliferation [3] and very little amount of IFN-γ secretion in the presence of CEA_99–111_, while no significant production of IL-5 and GM-CSF was observed in the presence of any peptide (Fig. 5, *right panel* *s, grey bars*). IL-13 release was not tested. Cytokine treatment strongly augmented (23-fold increase) IFN-γ release against CEA_99–111_, while, as expected, no effect on IL-5 and GM-CSF production was observed (Fig. 5, *right panel* *s, black bars*). In both cases, the two cytokines induced modulation of Th2 or amplification of Th1 responses that were already present, although at very low level, in the absence of the cytokines combination, while there was no evidence of *de-novo* specific recognition of CEA peptides.

Collectively, these results suggest that IL-12 and IL-27 promote functional modulation of Th2-type and amplification of pre-existing Th1-type anti-CEA responses.

## Discussion

In the present study, we report that the association of the immunomodulating cytokines IL-12 and IL-27 is able to modulate the functional polarization of anti-CEA Th2 CD4^+^ T cells from PC patients and to enhance pre-existing Th1 type anti-CEA CD4^+^ T cells.

We show that treatment with IL-27 as a single agent inhibited both IL-5 and IL-13 release by CEA-specific Th2 cells. This confirms in the human system a previous report that described IL-27 as able to suppress Th2 cytokines production from *in vitro* polarized mouse Th2 cells [12]; but, at difference with this report, in our system in which we deal with *bona fide in vivo* polarized CD4^+^ T cells IL-27 had no effect on IFN-γ secretion. Importantly, we also revealed a new function for IL-27 that is the inhibition of GM-CSF production. When IL-12 was used as a single agent, it strongly enhanced IFN-γ production, while had only a marginal effect in suppressing the release of Th2 cytokines, and of IL-13 in particular and no effect on GM-CSF. This is also at difference with previous reports [7], [8], in which IL-12 was shown to revert polarization of human allergen specific Th2 cells by inducing IFN-γ and inhibition of IL-4 production. Indeed, in the case of our clones IL-4, which however was not produced in high amounts, was not inhibited neither by IL-27 nor IL-12. The only Th2 cytokine affected by IL-12 was IL-5; this function was also previously reported for T cell clones obtained from the bronchoalveolar lavage of asthmatic patients [19].

Collectively, we show that in our system IL-12 and IL-27 have non redundant roles in modulating the polarization of established CEA-specific Th2 CD4^+^ T cells and that modulation of the functional and phenotypic Th2 polarization of anti-tumor CD4^+^ effectors into a Th0 type was obtained only in the presence of the combined synergistic treatment with the two cytokines. Modulation of the cytokines' production was, at least in the case of the clones, mostly functional with the induction of a rather peculiar phenotype where CCR4, CRTH2 and CCR5 expression coexisted (*i.e.*, not typical of either Th1 or Th0 cells). Preliminary experiments (data not shown), in which we evaluated cytokines' production at single cell level by intracellular staining, indicated that: *i*) IL-27 alone did not affect the percentage of IL-5 and IL-13 or IFN-γ producing cells both as single positive and double positive; and *ii*) IL-12 alone or the combination of the two cytokines induced a percentage of CD4^+^ T cells producing both Th2 cytokines and IFN-γ without reduction in the number of CD4^+^ T cells producing Th2 cytokines. These results along with the previous ones on cytokine secretion in the supernatants exclude that the effect of IL-12 on IFN-γ up-regulation is due to a subpopulation of Th1 cells that becomes preferentially expanded and suggest that the combination of IL-27 and IL-12 induces a reduction of Th2 cytokines secretion per single cell and reverts a small population of CD4^+^ Th2 cells, which then produce also high levels of IFN-γ, to a Th0 type. Further experiments are needed to better define the mechanism of action of the two cytokines both as single agent and in combination.

The effects of modulation of Th2 polarization seemed more effective for CEA-specific polyclonal CD4^+^ T cells where combined treatment was able to almost completely abolish IL-13 and GM-CSF production (Fig. 5). Although, we cannot exclude that in the latter case treatment with the cytokines might have affected not only the effectors but also the APC, which in turn might have further changed the cytokine milieu. To address this issue future studies should compare gene expression of different APC at basal level and after modulation with different cytokines' combination.

There are two main positive effects correlated to the modulation of the Th2 and enhancement of Th1 polarization of CEA-specific CD4^+^ T cells in PC. First, on the anti-tumor immunity: indeed, in human tumors a skew towards Th2 type of tumor-specific CD4^+^ T cells has been described in patients with advanced malignancies [20], [21], [22], while high levels of infiltrating Th1 cells in the tumors correlate with a better prognosis [23]. Second, on the tumor microenvironment, as fibrinogenesis and recruitment of myeloid suppressor cells are potentially inhibited. Indeed, Th2 cytokines and IL-13 in particular are strongly associated with stroma deposition, which is a peculiar feature of PC and is potentially involved in tumor progression, while IFN-γ suppresses collagen synthesis by fibroblasts [4]. Furthermore, high levels of GM-CSF in anti-tumor vaccines enhance the recruitment of myeloid suppressor cells, thus potentially favoring immune-escape of the tumor [24], [25].

Overall, our results demonstrate that *in vivo* polarized Th2 CD4^+^ T cells specific for a tumor-associated antigen from PC patients are endowed with plasticity, and support further studies aimed at investigating the possible clinical application of the combination of IL-12 and IL-27 as a treatment for pancreatic cancer both as cytokines delivery and possibly for reprogramming tumor antigen specific T cells prior to adoptive immunotherapy.

Treatment with systemic IL-12 of mouse cancer models has been shown to have a strong anti-tumor effect [26], [27]. However, systemic administration of IL-12, although stimulated anti-tumor immunity, had only minimal clinical efficacy in the presence of relevant side effects [28], [29]. Attempts to achieve IL-12 production only at the tumor site in melanoma and gastrointestinal carcinomas resulted instead in the absence of significant toxicity and in increased clinical responses, which correlated with increased inflammation [30], [31], [32]. In mouse models also IL-27 had a strong anti-tumor effect, which was enhanced by the presence of IL-12 [33], [34], [35], [36].

To avoid systemic toxicity, strategies to selectively deliver various therapeutic compounds, including IL-12, to the tumor by tumor-specific antibodies or to the tumor vasculature have been recently developed [37], [38]. Of note, some of these strategies are directed towards tenascin, which is a component of the extracellular matrix and it is produced by pancreatic stellate cells, possibly under the influence of soluble factors released by tumor cells [39]. Therefore, tenascin could represent an interesting target for tumor-delivery of IL-12 and IL-27 in PC.

The advantages of tumor targeted delivery of combined IL-12 and IL-27 in PC would be the modulation of the tumor microenvironment not only through their known effect on resident antigen presenting cells (and therefore on the *de-novo* priming of new Th1 effectors in the draining lymph nodes) but also directly on the modulation of the cytokines secreted by Th2 GATA-3+ infiltrating T cell effectors already present in the tumor by diminishing their negative effects on fibrinogenesis and recruitment of immunosuppressive myeloid dendritic cells, in the absence of systemic toxicity.
